# 

**Published:** 1876-05

**Authors:** 


### CONTENTS:

### ORIGINAL COMMUNICATIONS.

ARTICLE I.—Notes on the American Dental Association, 1875. By S. P. Cutler, M. D., D. D. S. .......... 1

ARTICLE II.—Artistic Knowledge and Critical Taste in Dentistry. By George H. Perine, D. D. S. .......... 7

ARTICLE III—Dental Education. By. W. C. Barrett, D. D. S. .......... 12

Georgia State Dental Society .......... 17

### SELECTED ARTICLES.

ARTICLE IV—A Portion of a Tooth Imbedded in Lower Lip. By Francis Mason, F. R. C. S. .......... 18

ARTICLE V.—The Air as an Anæsthetic. By W. G. A. Bonwill, D. D. S. .......... 23

ARTICLE VI.—The Eyes and the Teeth; Have They any Nervous Connection? By J. E. Cravens, D. D. S. .......... 28

ARTICLE VII.—The Effect of Medicines upon the Teeth. By J. F. Hodson, D. D. S 31

ARTICLE VIII.—Ether and Chloroform .......... 34

ARTICLE IX.—Dental Caries .......... 35

ARTICLE X.—The "Stuck Method" Simplified. By John D. Wingate, D. D. S. .. 37

ARTICLE XI.—The Use of Pestles and Mortars .......... 38

### EDITORIAL, ETC.

The Food we Eat .......... 39
A Dangerous Dentist .......... 41

### MONTHLY SUMMARY.

Chloral as an Anæsthetic .......... 42

Painless Opening of Abscesses .......... 42

The Blue Line in Lead Poisoning .......... 43

Resection of a Portion of Lower Jaw .......... 43

Topical Application in Painful Dentition .......... 44

Milk as a Vehicle of Contagion .......... 44

Avoidance of Phosphorous Poisoning .......... 45

Borax as an Antiseptic .......... 45

Necrosis of the Lower Jaw .......... 45

The Discovery of Anæsthesia .......... 46

To Check Epistaxis .......... 46

Female Students .......... 46

Medicated Ice .......... 47

Extracting Teeth for Brain Disease .......... 47

The Plethysmograph .......... 48

For Burns .......... 48

